# Reducing Addiction in Bipolar Disorder via Hacking the Dopaminergic System

**DOI:** 10.3389/fpsyt.2021.803208

**Published:** 2021-12-14

**Authors:** Heinz Grunze, Réka Csehi, Christoph Born, Ágota Barabássy

**Affiliations:** ^1^Psychiatrie Schwäbisch Hall, Schwäbisch Hall, Germany; ^2^Paracelsus Medical University, Nuremberg, Germany; ^3^Gedeon Richter Plc, Medical Division, Budapest, Hungary

**Keywords:** substance use disorder (SUD), cariprazine, psychopharmacotherapy, partial agonist, antipsychotic, bipolar disorder

## Abstract

The dopaminergic system plays a central and decisive role in substance use disorder (SUD), bipolar disorder (BD), and possibly in a subgroup of patients with refractory depression. Common genetic markers and underlying cellular processes, such as kindling, support the close link between these disorders, which is also expressed by the high rate of comorbidity. Although partial dopamine agonists/antagonists acting on D_2_ and D_3_ receptors have an established role in treating BD, their usefulness in SUD is less clear. However, dopamine D_3_ receptors were shown to play a central role in SUD and BD, making D_2_/D_3_ partial agonists/antagonists a potential target for both disorders. This narrative review examines whether these substances bear the promise of a future therapeutic approach especially in patients with comorbid BD and SUD.

## Introduction

Bipolar disorder (BD) is a complex and serious psychiatric disorder characterized by recurrent mood episodes. Its prevalence is estimated to be at least around 1% in the general population, and it is associated with premature death with a loss of 10–20 years of life attributable to both physical and psychiatric comorbidities (1). Its co-occurrence with other mental illnesses is the norm rather than the exception, especially with substance use disorder (SUD) (2).

## Prevalence

The prevalence of SUD in BD population was extensively explored by Hunt et al. (3, 4) who gathered data from clinical settings and national surveys conducted between 1990 and 2015. The prevalence of SUD was found to be more than 30% in community-, and more than 40% in clinical settings. Alcohol use disorder (AUD) was the most prevalent SUD with 20–30% prevalence rates in both community and clinical settings. Among illicit drugs, cannabis was the most commonly abused drug (around 20%), followed by cocaine (around 10%). The findings of these meta-analyses are in line with those of other studies with cannabis use ranking second after AUD (2, 5, 6).

## Significance and Consequences of Comorbidity

Both BD and SUD have been associated with detrimental consequences on their own, but the co-occurrence of SUD further complicates the already heterogenous clinical presentation of BD, often masking BD diagnosis and predicting an even worse prognostic outcome for patients (3, 7). Such patients experience more frequent and severe mood destabilizations, increased hospital admissions, accentuated depressive symptoms, an increased likelihood of suicidal behavior and suicide attempts as well as interference with the efficacy of therapeutic medications—either by lowering their mood stabilizing effects or requiring higher doses of the medication to achieve the therapeutic dose (3, 8, 9). Furthermore, earlier mean age of onset was observed for comorbid SUD in BD patients (20.7 years), compared to BD individuals without a lifetime prevalence of SUD (24.0 years), reflecting a significant difference in age of onset in these groups (3). Earlier onset of BD was found to result in a more severe course of illness (10).

## Shared Underlying Mechanisms and the Role of the Dopaminergic System and the D3 Receptors

As patients with comorbid SUD and BD present with accentuated severity of symptoms and have worse prognostic outcomes, shared underlying physiological mechanisms of these disorders are implied and several hypotheses were proposed in support of this notion (11).

One mechanism proposed to underlie SUD and BD comorbidity involves “kindling” which refers to the concept that neurons become increasingly sensitized due to repeated disruptions—and increased sensitization makes them more susceptible to interruption (12). Sensitization is observable in both SUD, where individuals progress from occasional to frequent substance use, and BD, where mood becomes increasingly unstable, depressive, and manic episodes alternate with greater frequency and intensity and periods of remission become briefer (13). Thus, the notion of kindling holds that some individuals might be more vulnerable to neuron sensitization, increasing their risk for developing both SUD and BD.

Furthermore, genetic risk factors are known to play a role in the development of both SUD and BD. Individuals with SUD have a greater chance of having a family member with mood disorder than individuals without such family members—and vice versa—suggesting that SUD and BD might share common gene variants that increase the risk for developing both disorders (12).

The “disorder fostering disorder” concept suggests that the pathological effects of BD and SUD might increase the risk for developing the other (12). Patients with BD might look to self-medicate in order to alleviate their symptoms by taking drugs or consuming alcohol. This view implies that having BD increases the risk for developing SUD. However, the reverse is also true, as substance use exacerbates pathophysiological changes in the already dysfunctional neurotransmitter systems or signaling pathways (14).

The concept of allostasis (the process of maintaining homeostasis through the adaptive change of the organism's internal environment) may provide further insights in the understanding of the pathogenetic mechanisms underlying the comorbidity of BD and SUD (15): if BD is assumed to be a disease involving the cumulative build-up of allostatic states, which as a progressive dysregulation of reward circuits is expressed as negative affective states, it may leave BD patients more vulnerable to drug addiction (16). Furthermore, functional neuroimaging studies identified abnormalities of brain networks—the Default Mode Network—in BD and SUD that are possibly involved in the pathophysiology of both disorders and therefore provide evidence for the shared underlying mechanisms (15).

Yet another mechanism proposed to underlie SUD and BD comorbidity concerns the role of the dopaminergic system, which was recognized a long time ago in both BD and SUD. In BD, bipolar depression is characterized by increased striatal dopamine transporter levels, resulting in attenuated dopaminergic function (17). In contrast, increased D_2_/D_3_ receptor availability as well as hyper-responsive reward system in the ventral striatum is observed in bipolar mania, leading to heightened dopaminergic neurotransmission (17). In SUD, nearly all neurochemical systems in the brain are involved in the pathophysiology, including the dopaminergic system which has been extensively examined due to its involvement in reward and reinforcement (18). Particularly the D_3_ receptor system and its significance in addiction sparked interest: firstly, due to its anatomical localization, as D_3_ receptors are highly expressed in limbic areas that form the “reward” circuitry, therefore implying that they mediate motivation, emotions, and by extension, may be involved in addiction (19). The other pivotal feature of D_3_ receptors is that they have the highest overall affinity to endogenous dopamine (Ki = 30 nM) among the five dopamine-subtypes (20, 21). Thus, they are the most sensitive to basal concentration (19), indicating greater occupancy of D_3_ receptors after dopamine-elevating drug administration (most drugs of abuse) in comparison with D_1_ or D_2_ receptors (estimated to be 96% vs. 25–27%) (22).

Human positron emission tomography (PET) studies have contributed greatly to bringing light to the dopaminergic abnormalities in addictions, especially related to the D_2_-like dopamine receptors (D_2_ and D_3_), by allowing measurement of receptor occupancy (18). Reduced striatal D_2_ receptor availability was found in individuals with SUD [including cocaine (23), alcohol (24), and methamphetamine (25)] compared to healthy controls (18). These abnormalities have been linked to behavioral traits relevant to addiction, such as emotional and behavioral impulsivity (26)—which is also a common feature in BD—, but also in response inhibition (27) and relapse after clinical intervention (28). PET studies further discovered blunted dopamine release at D_2_ receptors in subjects with addiction [including cocaine (29), alcohol (24), and methamphetamine (25)], assumed to be associated with hypoactive dopaminergic state that bolsters drug-seeking behavior (18).

Recent findings, however, have found that unlike D_2_ receptors, D_3_ receptors have actually shown an upregulation in human *post-mortem* (30) and animal studies (18, 31). Despite these *in vitro* findings, the examination of D_3_ receptors in humans *in vivo* was restricted due to the lack of a selective PET ligand. The relatively recent introduction of [^11^C]-(+)-PHNO—a D_3_ preferring PET radioligand—has, however, enabled the investigation of D_3_ receptors in addiction in the human brain *in vivo* (19, 32). Indeed, PET studies using [^11^C]-(+)-PHNO confirmed the findings of *in vitro* studies: D_3_ receptor availability is heightened in individuals with SUD, and they were shown to be associated with impulsivity (23), drug craving (33), cognitive dysfunction (34), and symptom severity (18).

Thus, evidence suggests that both SUD and BD share similar dopaminergic dysfunctions especially at the D_2_ and D_3_ receptors, which shifts the attention toward dopamine modulating agents such as partial agonists acting at the D_2_/D_3_ dopamine receptors.

## Treatment of BD and SUD

Traditionally, comorbid SUD in BD or other psychiatric illnesses have usually been treated either in parallel, i.e., patients were receiving concurrent treatment for both disorders, but in different programs, or in sequence, i.e., SUD first, BD second (9). Despite extensive evidence highlighting the frequency of the occurrence of SUD in BD, as well as its detrimental impact on the prognosis and treatment outcomes of BD, only a few studies aimed at exploring appropriate treatment options for this subgroup of BD patients, especially in terms of pharmacotherapies (9). Instead, BD has been traditionally treated with mood stabilizers and anticonvulsant agents, or with second-generation antipsychotics (35). For SUD, the need for pharmacological therapy has long been acknowledged, yet adequate therapeutic options are lacking (36). Current medications include (depending on the substance of abuse) buprenorphine, naltrexone, topiramate, varenicline, bupropion, clonidine, and methadone (37). Given antipsychotics' dopamine-stabilizing effects, they were anticipated to reduce craving in SUD, leading researchers to investigate this notion (2). According to a meta-analysis, the antipsychotics investigated in the study (amisulpride, aripiprazole, olanzapine, and quetiapine) did not produce significant reductions in alcohol craving or drinking behavior in patients with primary AUD without comorbidities (38). Aripiprazole, however, was significantly associated with a decrease in the number of drinks as well as heavy drinking days (39). Furthermore, a study involved patients with comorbid BD/schizoaffective disorder and SUD who were switched to aripiprazole (40). Patients with comorbid AUD showed reduced alcohol craving and spent less dollars on alcohol, while patients with cocaine use disorder showed a decrease in cocaine craving, but not cocaine use (40). Quetiapine, an atypical antipsychotic with a very low affinity for D_2/3_ receptors (41), was further investigated whether it relieves alcohol craving similarly to aripiprazole (42). Results, however, did not demonstrate efficacy in a randomized controlled trial for alcohol use measures in patients with comorbid BD and AUD (42).

In light of the shared underlying mechanisms, integrated treatment options—addressing both disorders by the same team at the same time—need to be established for this patient population (9), and the most likely drug candidates to treat with seem to be partial agonists acting at D_2_/D_3_ dopamine receptors.

## Dopamine D_2_/D_3_ Partial Agonists in the Treatment of BD and SUD

The currently known and markedly available dopamine D_2_/D_3_ partial agonists are aripiprazole, cariprazine, and brexpiprazole (43). Some older compounds (such as bifeprunox), as well as some newer compounds in development (e.g., OSU-6162) also exist and provide valuable information to the understanding of the efficacy of partial agonists in BD and SUD (44, 45).

The efficacy of D_2_/D_3_ partial agonists in SUD is not well-examined and much of the data comes from animal studies. As such, one animal model has investigated the anti-abuse effects of cariprazine, aripiprazole, and bifeprunox in cocaine addiction in rats (44). All compounds succeeded at reducing the rewarding effects of cocaine—as indicated by enhanced self-administration of the drug—as well as prevented relapse to cocaine seeking following a period of complete withdrawal from cocaine and its related cues (44). Equipotent effects of cariprazine and bifeprunox were observed, 20 times more potent than that of aripiprazole (44). The beneficial effects of partial agonists in animal studies were also observed in alcohol abuse: the compound OSU-6162 effectively reduced self-administration, withdrawal and reinstatement in rats (45), and aripiprazole lessened the acute stimulant effects of alcohol in mice (46, 47). Furthermore, one study investigated the effect of a D3 partial agonist, CJB090, in methamphetamine addiction in rats, where the investigational drug yielded reductions in methamphetamine self-administration (fixed ratio schedule) and its excessive intake in a group of rats with extended access to methamphetamine (48). Human data in SUD is scarce, and little information is available.

**Figure 1:**
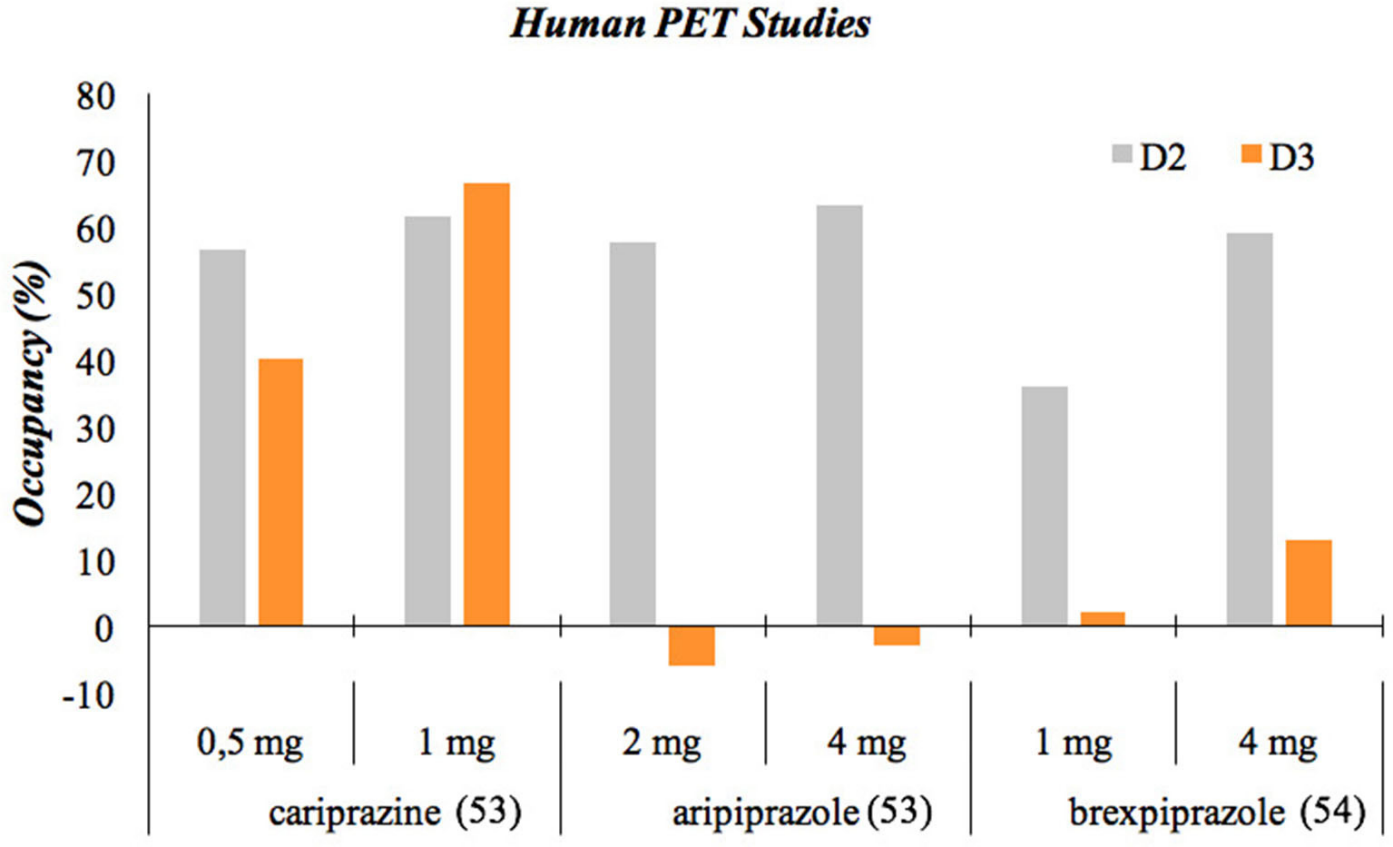
Occupancy of D_2_ and D_3_ receptors of antipsychotics. The cariprazine and aripiprazole data come from a PET study involving healthy volunteers that aimed to assess the D3 receptor occupancy of cariprazine and aripiprazole at doses that attain similar D2 receptor occupancy by both drugs. This was assessed using two different methods, and the data presented here is the average of the outcome of these methods. The brexpiprazole data comes from a PET study involving schizophrenia patients.

The efficacy of D_2_/D_3_ partial agonists in BD has been well-examined for the currently available compounds with different findings. While cariprazine proved to be efficacious in both bipolar mania and bipolar depression (49) [3–6 mg in bipolar mania (50) and 1.5–3 mg in bipolar depression (51)], studies of aripiprazole confirmed efficacy in bipolar mania only (52). Brexpiprazole studies in bipolar mania were unsuccessful (53), and, following a positive pilot trial (54), a RCT in bipolar depression is ongoing (55). Human PET studies with cariprazine (56, 57), aripiprazole (58), and brexpiprazole (58) have pointed to the difference potentially explaining these findings: while all three compounds were able to occupy the D_2_ receptors in the brain, only cariprazine was able to sufficiently occupy the D_3_ receptors as well [(59); Figure 1]. Additionally, a clinical trial has been initiated to further study the dopamine D3 receptor occupancy of cariprazine (1.5 vs. 3 mg/day) in patients with unmedicated bipolar depression (60).

Cariprazine has in fact a preferential binding to D_3_ receptors, and its binding is stronger than that of any other antipsychotics and even dopamine itself (61). Given dopamine's very high affinity for the D_3_ receptors, the low affinities of antipsychotics, with the exception of cariprazine, make them unable to block the D_3_ receptors in the presence of dopamine in the living brain (62). This means that only cariprazine is able to exhibit the effects usually associated with D_3_ partial agonism, which are improvements in negative, cognitive and depressive symptoms as well as in motivation and reward (49). Given the increasingly acknowledged role of D_3_ in SUD along with BD, cariprazine's high affinity for D_3_ receptors makes it an appropriate candidate for the treatment of comorbid BD with SUD.

Two clinical trials have been initiated to investigate cariprazine's efficacy in SUD, although results are not available yet. An investigator-initiated trial aims to explore cariprazine's effects on the brain and behavior in cocaine use disorder in a phase II, randomized, single-blind, placebo-controlled study using fMRI (1.5 vs. 3 mg/day) (63). Furthermore, a phase IIa, randomized, placebo-controlled pilot study was designed to explore how low-dose cariprazine (1.5 mg/day) affects cocaine use in medically stable patients with comorbid opioid use disorder who have already been taking buprenorphine/naloxone at a stable dose (64). Additionally, scarce data is available from case reports as summarised in Table 1. Evidence for the effects of several partial agonists in SUD, BD, and BD or related psychotic disorders and comorbid SUD is depicted in the Supplementary Table 1, which also includes two additional recent case reports on cariprazine treatment in major psychiatric disorders with comorbid SUD (67).

**Table 1:** Cariprazine case reports.

Sanders and Miller (65)	
Age	51
Gender	Male
Problem	Bipolar I disorder with alcohol use and cocaine craving
Cariprazine's effect	Reduced substance use, craving, and improved mood symptoms
Short description	The patient had failed multiple medication trials (including risperidone, paliperidone, aripiprazole, bupropion SR, carbamazepine, lamotrigine, and lithium) for treatment of bipolar I disorder symptoms. When he got enrolled in a cariprazine (monotherapy) trial, he was suffering from alcohol abuse and craving cocaine. The transformation of his appearance and presentation was remarkable. He seemed well-groomed unlike during the previous appointments, as well as he reported a lowered urge to drink excessively or use drugs and he was in a stable mood. He stopped using illicit drugs and his drinking behavior has continuously declined, he is now abstinent
Age	20
Gender	Female
Problem	Bipolar I disorder, ADHD, alcohol, and cannabis use
Cariprazine's effect	Improved mood and behavior symptoms, reduced substance use, enhanced overall functioning
Short description	Besides the bipolar I disorder diagnosis, the patient suffered from ADHD, alcohol, and cannabis use as well. Several medications had been tried to mitigate her symptoms of depression, irritability, distractibility, and agitation with little success Cariprazine was started as an add-on treatment at 1.5 mg/day for 3 weeks without improvement; then it was increased to 3 mg/day. Her medication regimen at that time included quetiapine 25 mg/day at bedtime, clonazepam 0.5 mg twice daily at bedtime, and methylphenidate XR 72 mg daily. After 3 weeks on 3 mg cariprazine, she presented with significant improvement—no restlessness, good eye contact, organized thought processes, respectful of her mother's input, and most remarkably she was substance-free. She agreed to random urine toxicology screens, both of which were negative. Following several months of substance abstinence and respectful behavior toward her family, her parents allowed her to return to live at their home. She has been free of substance abuse for 27 months, she continues to function well, her symptoms remain improved, and she recently graduated at the top of her class in an aesthetician training program and has passed all of her state boards
Age	54
Gender	Male
Problem	Bipolar I disorder, alcohol use
Cariprazine's effect	Improved mood and behavior symptoms, reduced substance use, and enhanced overall functioning
Short description	Although the patient and his wife run their own business, he was functionally disabled by his comorbid bipolar I disorder and alcohol use disorder. At the time of his initial presentation, he was taking quetiapine, lithium, lamotrigine, bupropion, duloxetine, omega-3 fatty acids, and gabapentin. Subsequent medication trials included various combinations of lurasidone, olanzapine, methylphenidate, and asenapine. Although there was some benefit for his depression, his excessive alcohol use persisted. After the initiation of cariprazine as add-on treatment to his current regimen, he reported a dramatic decline in alcohol-craving and eventually restricted his alcohol intake to 1–2 drinks on holidays or special occasions only. He was then tapered off his previous medications, and he is now stable and functioning well on cariprazine and quetiapine
Ricci et al. (66)	
Age	21
Gender	Male
Problem	Methamphetamine-induced psychosis
Cariprazine's effect	Improved mood and behavior symptoms, reduced substance use, and enhanced overall functioning
Short description	The patient progressed from occasional methamphetamine use at the age of 23 to daily use by the age of 24. He was admitted to the hospital after presenting with persistent visual and auditory hallucination, suspiciousness and social withdrawal, with symptoms remaining after ceasing methamphetamine use. He developed depressive, negative, and cognitive symptoms and suicidal thoughts. After his hospital admission, he received olanzapine with no improvement, followed by risperidone which improved depressive symptoms. He then received cariprazine (starting dose of 1.5 mg/day for 3 days, then 3 mg/day between day 4 and 12, then 4.5 mg/day from day 13 onwards) and benzodiazepines for insomnia. Two weeks of cariprazine treatment yielded an improvement in paranoid and hallucinatory symptoms, and in social functioning, resulting in his discharge. At week 16 of his treatment, his scores on the negative and positive subscales of the Positive and Negative Syndrome Scale (PANSS) were reduced by 61.7 and 69.9%. The patient regained his baseline level of social and occupational functioning, and reported a decrease in methamphetamine use and craving. Cariprazine dose was then reduced to 3 mg/day, and the improvement in symptoms was maintained during the treatment period. The patient remains on cariprazine monotherapy and during the treatment period, he remained free of psychotic symptoms and abstinent from methamphetamine

## Conclusion

So far, pharmacological treatment concepts hardly considered the joint treatment of SUD and BD, which seem to share a common action on dopamine D_2_ and D_3_ receptors. An ideal integrated pharmacological treatment would therefore address both disorders through the D_2_ and D_3_ receptors, in addition to other therapeutic interventions, such as psychotherapy. Since cariprazine has shown to exert effects on both D_2_ and D_3_ receptors (partial agonist effect) next to serotonin receptors, as well as has well-established efficacy in bipolar I disorder, it is believed to be a potential treatment option for this patient population. Data for this assumption comes from animal studies and case reports, however, further studies are needed to validate this rationale-based assumption.

## Author Contributions

HG, RC, CB, and ÁB contributed to developing the concept of the manuscript. RC wrote the first draft of the manuscript. All authors contributed to manuscript revision, read, and approved the submitted version.

## Funding

Gedeon Richter provided funds for the open access publication fees.

## Conflict of Interest

RC and ÁB are employees of Gedeon Richter Plc. The remaining authors declare that the research was conducted in the absence of any commercial or financial relationships that could be construed as a potential conflict of interest.

## Publisher's Note

All claims expressed in this article are solely those of the authors and do not necessarily represent those of their affiliated organizations, or those of the publisher, the editors and the reviewers. Any product that may be evaluated in this article, or claim that may be made by its manufacturer, is not guaranteed or endorsed by the publisher.
