# Supplementary material for: Reducing Addiction in Bipolar Disorder via Hacking the Dopaminergic System
Source: Front Psychiatry. 2021 Dec 14;12:803208. doi: 10.3389/fpsyt.2021.803208 (PMC8712474; doi:10.3389/fpsyt.2021.803208)
Supplement: Supplementary file 1 [file Table_1.docx]

Supplementary Table 1: Evidence cited in the article for the efficacy of several partial agonists in SUD, BD, and BD or related psychotic disorders and comorbid SUD.

|  | **Type of study** | **Problem/disorder** | **Investigational drug** | **Outcome** |
| --- | --- | --- | --- | --- |
| Román et al., 2013 | Animal study (rats) | Cocaine abuse | Cariprazine  Aripiprazole  Bifeprunox | All compounds reduced the rewarding effects of cocaine and prevented relapse. Cariprazine and bifeprunox had equipotent effects, 20x more potent than aripiprazole |
| Steensland et al., 2012 | Animal study (rats) | Alcohol abuse | OSU-6162 | Reduced self-administration, withdrawal and cue-induced reinstatement |
| Jerlhag et al., 2008 | Animal study (rats) | Alcohol abuse | Aripiprazole | Lessened the acute stimulant effects of alcohol without interfering with general motor activity |
| Orio et al., 2010 | Animal study (rats) | Methamphetamine abuse | CJB-090 | Reduced self-administration and excessive intake |
| Ketter et al., 2018 | Clinical trial | BD mania | Cariprazine | Effective |
| McIntyre et al., 2020 | Clinical trial | BD depression | Cariprazine | Effective |
| Li et al., 2017 | Clinical trial | BD mania | Aripiprazole | Effective |
| Li et al., 2017 | Clinical trial | BD depression | Aripiprazole | Not effective |
| Vieta et al., 2021 | Clinical trial | BD mania | Brexpiprazole | Not effective |
| NCT04569448 | Clinical trial | BD depression | Brexpiprazole | Clinical trial is ongoing |
| NCT03430544 | Clinical trial | Cocaine use disorder | Cariprazine | Clinical trial is ongoing |
| NCT05063201 | Clinical trial | Cocaine and opioid use disorder | Cariprazine add-on to buprenorphine/naloxone | Clinical trial is ongoing |
| Sanders & Miller, 2019 | Case report | BD with cocaine and alcohol abuse | Cariprazine | Improved mood symptoms and led to substance abstinence in a 51-year-old male patient. |
| Sanders & Miller, 2019 | Case report | BD and ADHD with alcohol and cannabis abuse | Cariprazine add-on | Improved mood and behavioural symptoms, led to substance abstinence, enhanced overall functioning in a 20-year-old female patient |
| Sanders & Miller, 2019 | Case report | BD with alcohol abuse | Cariprazine add-on | Improved mood and behaviour symptoms, reduced substance use, and enhanced overall functioning in a 54-year-old male patient. |
| Ricci et al., 2021 | Case report | Methamphetamine-induced psychosis | Cariprazine | Improved mood and behavioural symptoms, led to the discontinuation of methamphetamine use, and enhanced overall functioning in a 21-year-old male patient. |
| Troung & Li, 2021 | Case report | Methamphetamine use disorder, methamphetamine-induced psychosis and PTSD, tobacco use disorder and opioid use disorder on buprenorphine/naloxone | Cariprazine | Reduced methamphetamine craving (confirmed by negative urine tests), led to a desired loss of weight gained from other antipsychotics, improved sleep and global functioning in a 33-year-old male patient |
| Troung & Li, 2021 | Case report | Methamphetamine use disorder, stimulant-induced psychotic disorder, PTSD, bulimia nervosa, tobacco use disorder and opioid use disorder on buprenorphine/naloxone | Cariprazine | Reduced methamphetamine craving and eventually discontinuation, stabilised weight and sleep, and improvement of hypervigilance in a 51-year-old transgender male to female patient |
